# Supplementary material for: Determinants of preterm birth among women delivered in public hospitals of Western Ethiopia, 2020: Unmatched case-control study
Source: PLoS One. 2021 Jan 25;16(1):e0245825. doi: 10.1371/journal.pone.0245825 (PMC7833256; doi:10.1371/journal.pone.0245825)
Supplement: S1 Questionnaire — (DOCX) [file pone.0245825.s001.docx]

**Wollega university**

**Institute of health sciences**

**Consent form**

This questionnaire is prepared by Wollega university, School of nursing & midwifery research team to conduct the study on “**Determinants of preterm birth among women delivered in public hospitals of Western Ethiopia, 2020: Unmatched case-control study”.** You have been included randomly to this study and the information you will provide will neither be disclosed to the third party, nor be used for another purpose and will be rather kept confidential. The information collected will not be linked to you and you don’t need to tell your name. You have full right to participate, reject or not to answer all or part of the questions. However, your participation in giving the right information is quite important for our study.

Do you agree to participate?

Yes Continue your interview

No Thank him or her

Name of interviewer/data collector

Signature

Crosschecked by the supervisor A/yes B/No Signature

**Questionnaires**

**Part I: Sociodemographic characteristics of the mothers**

1. What is your age in years? _____________
2. What is your age at first marriage in years? ___________
3. What is your Ethnicity?
4. Oromo B. Amhara C. Gurage D. Tigre E. Others
5. What is your religion?
6. Protestant B. Orthodox C. Catholic D. Muslim E. Others
7. What is your current marital status?
8. Married B. Single C. Divorced D. Widowed
9. What is your highest educational status?
10. Unable to read and write C. Completed grade 9-12
11. Completed grade 1-8 D. Diploma and above
12. What is your current occupation?
13. Government employee D. Farmer E. Others
14. Private employee E. Merchant
15. What is your husband’s current occupation?
16. Government employee C. Merchant E. Others
17. Private employee D. Farmer
18. What is your residence?
19. Urban B. Rural
20. What is your family size in number? ______________
21. What is your family’s average monthly income in birr? ______________
22. Time to reach nearby health facility in hour ___________

**Part II: Obstetrics related characteristics of the mothers**

1. How many times you gave birth including the current birth?

A. 1^st^ pregnancy

B. 2 times

C. 3 times

D. 4 times

E. > 4 times

2. What is the duration between the current & previous childbirth in years?

A. < 2 years

B. ≥ 2 years

3. Did you or your partner use family planning before this current pregnancy?

A. Yes B. No

4. Did you plan this pregnancy?

A. Yes B. No

5. What was your status of antenatal care attendance for the current pregnancy?

A. Not attended at all

B. 1 time

C. 2 times

D. 3 times

E. ≥ 4 times

6. What is the mode of delivery for the current pregnancy?

A. SVD C. CS E. Destructive delivery

B. Forceps D. Vacuum delivery

7. Have you had history of previous preterm birth?

A. Yes B. No

8. Have you experienced premature rupture of membrane in this current pregnancy?

A. Yes B. No

9. How is your current labor started?

A. Spontaneous B. Induced

10. Have you had history of abortion?

A. Yes B. No

11. What is your current number of child born?

A. Singleton B. Multiple

**Part III: Medical history related characteristics of mothers**

1. Have you had a history of diabetes mellitus during the current pregnancy?
2. Yes B. No
3. Have you had a history of cardiac disease during the current pregnancy?
4. Yes B. No
5. What is your HIV/AIDS status?
6. Positive B. Negative C. Unknown
7. Have you experienced anemia during the current pregnancy?
8. Yes B. No
9. Have you experienced malaria during the current pregnancy?
10. Yes B. No
11. Have you a history of Sexually transmitted diseases during this current pregnancy?
12. Yes B. No
13. Have you developed hypertensive disorders during the current pregnancy?
14. Yes B. No

**Part IV: Social, behavioral and nutritional related characteristics of mothers**

1. Have you had history of substance use?
2. Yes B. No
3. Did you get dietary supplementation during this current pregnancy?
4. Yes B. No
5. Maternal MUAC measurement in centimeters ______________
6. Did you get family/social support during the current pregnancy?
7. Yes B. No
8. Have you experienced physical abuse during this pregnancy?
9. Yes B. No
10. Have you ever used traditional medicine?
11. Yes B. No
12. Have you experienced any type of stress during the current pregnancy?
13. Yes B. No

**Part V: Preterm birth assessment (To be filled from cards of newly born infants)**

1. Is the newly born infant is delivered before 37 completed weeks of gestation?
2. Yes (Preterm) B. No (Term)

THANK YOU VERY MUCH!
