# Supplementary material for: Determinants of preterm birth among women delivered in public hospitals of Western Ethiopia, 2020: Unmatched case-control study
Source: PLoS One. 2021 Jan 25;16(1):e0245825. doi: 10.1371/journal.pone.0245825 (PMC7833256; doi:10.1371/journal.pone.0245825)
Supplement: Attachment — Submitted filename: Response to reviewers.docx [file pone.0245825.s003.docx]

**Point by point response**

**Editor comments:**

We encourage you to let the manuscript be checked for proof-reading by a native English speaker.

**Response:** Thank you very much for your suggestion to improve our manuscript. Based on your recommendation, we contacted native English speaker to correct the grammar, and we hope this version of our manuscript is now suitable for publication.

**Reviewer comments:**

Reviewer #2: Manuscript number: PONE-D-20-17494R1

This paper has been greatly improved and almost all my concerns have been addressed. However, the manuscript has still certain glaring errors. For example, line 25-26 “…1 million death…and 60% of this death occur in ….” has to be corrected as …1 million deaths… and 60% of these deaths occur in… Therefore, the manuscript needs to be revised for language thoroughly.

**Response:** Thank you very much once again for your great contribution in the improvements of this manuscript. The previous version of our manuscript has some grammatical and editorial problems. In this revised submission, all authors intensively reviewed the document and corrected grammatical and editorial problems. We also used free online grammar correction to solve this problem and contacted native English speaker to correct the grammar, and we hope this version of our manuscript is now suitable for publication.

Thank you very much
